# Pyruvate dehydrogenase kinase 1 is essential for transplantable mouse bone marrow hematopoietic stem cell and progenitor function

**DOI:** 10.1371/journal.pone.0171714

**Published:** 2017-02-09

**Authors:** Camilla Halvarsson, Pernilla Eliasson, Jan-Ingvar Jönsson

**Affiliations:** 1 Department of Clinical and Experimental Medicine, Linköping University, Linköping, Sweden; 2 Linköping Integrative Regenerative Medicine Centre, Linköping University, Linköping, Sweden; Emory University, UNITED STATES

## Abstract

**Background:**

Accumulating evidence suggests that hypoxic areas in the bone marrow are crucial for maintenance of hematopoietic stem cells (HSCs) by supporting a quiescent state of cell cycle and regulating the transplantation capacity of long-term (LT)-HSCs. In addition, HSCs seem to express a metabolic profile of energy production away from mitochondrial oxidative phosphorylation in favor of glycolysis. At oxygen deprivation, hypoxia inducible factor 1α (HIF-1α) is known to induce glycolytic enzymes as well as suppressing mitochondrial energy production by inducing pyruvate dehydrogenase kinase 1 (*Pdk1*) in most cell types. It has not been established whether PDK1 is essential for HSC function and mediates hypoxia-adapting functions in HSCs. While the *Pdk* gene family contains four members (*Pdk1-4*), it was recently shown that *Pdk2* and *Pdk4* have an important role in regulating LT-HSCs.

**Principle findings:**

Here we demonstrate that PDK1 activity is crucial for transplantable HSC function. Whereas *Pdkl*, *Pdk2*, and *Pdk3* transcripts were expressed at higher levels in different subtypes of HSCs compared to differentiated cells, we could not detect any major differences in expression between LT-HSCs and more short-term HSCs and multipotent progenitors. When studying HIF-1α-mediated regulation of *Pdk* activity *in vitro*, *Pdk1* was the most robust target regulated by hypoxia, whereas *Pdk2*, *Pdk3*, and *Pdk4* were not affected. Contrary, genetic ablation in a cre-inducible *Hif-1α* knockout mouse did not support a link between HIF-1α and *Pdk1*. Silencing of *Pdk1* by shRNA lentiviral gene transfer partially impaired progenitor colony formation *in vitro* and had a strong negative effect on both long-term and short-term engraftment in mice.

**Conclusions:**

Our study demonstrates that PDK1 has broad effects in hematopoiesis and is a critical factor for engraftment of both HSCs and multipotent progenitors upon transplantation to recipient mice. While *Pdk1* was a robust hypoxia-inducible gene mediated by HIF-1α *in vitro*, we could not find evidence of any *in vivo* links between *Pdk1* and HIF-1α.

## Introduction

Hematopoietic stem cells (HSCs) are located in the bone marrow (BM) where the balance between self-renewal and differentiation is under the influence of both cell-intrinsic and extrinsic signals. Numerous factors have been identified during the last decades that regulate HSCs including secreted factors and supportive cells such as endosteal, perivascular, endothelial, mesenchymal, and stromal cells [1, 2]. In addition, the BM is considered a relatively hypoxic tissue where HSCs reside mainly within niches of limited oxygen availability [3–5]. Cellular adaptation to hypoxia involves several important steps that regulate glucose metabolism, which serves to inhibit respiration and favoring energy production via glycolysis, thereby avoiding excessive mitochondrial oxidative phosphorylation (OXPHOS) [6] that otherwise would induce cycling and exhaustion of HSCs [7]. Multiple evidences suggest that HSCs are located to hypoxic BM regions. Therefore, it has been hypothesized that HSCs are dependent on such an environment of low O_2_. As a consequence HSCs would utilize anaerobic metabolism for proper regulation and maintenance [6, 8].

In many cell types, a low metabolic profile is primarily mediated by hypoxia-inducible factors (HIFs). HIFs are heterodimeric transcription factors consisting of two subunits; constitutively expressed HIF-1β [9] and either oxygen-sensitive HIF-1α or HIF-2α, which are degraded at the protein level when exposed to oxygen [10] but is stabilized at low levels of oxygen [9, 11, 12]. An important role of HIF-1α in hematopoiesis and HSC quiescence was first demonstrated in conditional *Hif-1α*^*-/-*^ mice, in which numbers of HSCs decreased when exposed to stress, such as aging or BM transplantation [13]. However, more recent studies suggest that HIFs are not indispensable for HSCs during steady-state *in vivo*. Thus, in different conditional knockout mice lacking either *Hif-1α*, *Hif-2α*, or both, or alternatively lacking *Hif-1β* impairing both HIF-1α and HIF-2α function, no clear evidence was provided for any short-term or long-term effects on the HSC compartment [14, 15]. Although these differences may be due to distinct mouse strains, HIFs may promote multiple functions in the BM and may be nonessential for proper HSC activity.

Repression of mitochondria function and oxygen consumption is regulated by HIF-1α-dependent activation of pyruvate dehydrogenase kinase 1 (*Pdk1*) [16, 17] or *Pdk3* [18], two members of the *Pdk* family [19, 20]. It was recently reported that all four *Pdk* family gene members are expressed in HSCs. Furthermore, *Pdk2* and *Pdk4* seem to be targets of HIF-1α as genetically modified mice lacking the *Hif-1α* gene have been shown to display reduced levels of both *Pdk2* and *Pdk4* [21]. In contrast, it has not been established whether PDK1 mediates hypoxia-adapting functions via induction by HIF-1α in HSCs. In the present study, we show that hypoxic exposure of Lineage^-^Sca1^+^c-kit^+^ (LSK cells) favors a switch away from mitochondrial OXPHOS to glycolysis by induction of genes encoding glycolytic enzymes and *Pdk1*. We demonstrate an important role of PDK1 in transplantable HSCs where gene silencing of *Pdk1* impaired the engraftment potential of both long-term (LT)-HSCs and multipotent progenitors (MPPs) upon transplantation to recipient mice. Compared with other *Pdk* gene family members, *Pdk1* was the main target of Hif-1α when determined *in vitro*. Contrary, we could not find any evidence for HIF-1α-dependent regulation of *Pdk1* in conditional *Hif-1α*^*-/-*^ mice.

## Materials and methods

### Animal ethics and housing

This study was reviewed and approved by the Linköping Animal Ethical Committee. Mice were bred and housed 4 per cage under conventional conditions in microisolator filter-top cages in the fully-accredited animal facility at Linköping University. Animal rooms were provided with 10–12 air changes per 24 hour, and maintained at 22 (± 2°C and a relative humidity of 50 (±20) %. Animals remained on regular 12-hour light-dark cycling, and received ad libitum food and acidified water.

For bone marrow transplantation, mice were acclimated 1–2 weeks before exposure to ionizing radiation (9 Gy) and subsequent injection of donor cells (maximum volume of 0.2 ml). After transplantation, mice were observed daily for 14-day post-irradiation and maintained under sterile conditions in microisolater filter-top cages and provided with autoclaved food and water containing 111mg/L ciprofloxacin (Ciproxin: Uppsala, Sweden). From day 15, mice were routinely monitored at least three times per week by trained animal technicians. An in-house scoring system was used to follow all irradiated mice and at first sign of illness (general appearance, reduced movement, ruffled fur, weight loss) animals were euthanized by CO_2_ inhalation followed by cervical dislocation. The transplantations included 76 mice of which 7 died due to effects of the lethal radiation dose of 9 Gy, 5 without euthanasia. Thus, the results in this study are based on 69 successfully transplanted mice.

### Mouse breeding and genotyping

*Hif-1α*^*flox/flox*^ (B6.129-Hif1a^tm3Rsjo^/J; Jackson Laboratory) mice [22] were mated to inducible *Mx1-Cre* mice to generate *Mx1-Cre*:*Hif-1α*^*flox/flox*^ mice. Offspring were genotyped by PCR-based assay with DNA from mouse ear snips (primers used are listed in S1 Table). To generate *Hif-1α*^*Δ/Δ*^ mice, the Cre transgene was induced by intraperitoneal injection of 400 μg poly I:poly C (pIpC; Sigma Aldrich, St Louis, MO) three times every 48 hours, into 7–12 week old mice. As a control, age-matched pIpC-treated *Hif-1α*^*+/+*^ mice were used.

### Isolation and culture of bone marrow cells

Femurs, tibiae, and iliac crests were dissected from sacrificed 8–12 weeks old mice and crushed in PBS supplemented with 5% heat-inactivated FBS (HyClone UK Ltd, Cramlington, UK) using a mortar and pestle. Bone fragments were removed from marrow by filtering through 70 Δm nylon mesh (Thermo Fisher Scientific, Asheville, NC). BM cells were enriched by positive selection for c-kit (CD117) expressing cells using immunomagnetic beads (Miltenyi Biotec, Bergisch Gladbach, Germany) before cell sorting. LSK cells were grown in StemSpan serum-free medium (Stem Cell Technologies, Vancouver, BC) with 50ng/mL of murine stem cell factor (SCF), human thrombopoietin (TPO), and human IL-6 (PeproTech Inc., Rocky Hill, NJ). LT-HSCs were grown in StemSpan Serum-free medium with 50ng/mL of murine SCF and human TPO, and MPPs with 50ng/mL murine SCF, human TPO and human FLT3. Before methylcellulose cultures, MPPs were grown in StemSpan serum-free medium with 50ng/mL murine SCF, human TPO, human IL-6 and human FLT3. Standard conditions for normoxia (20% O_2_) were 37°C in 5% CO_2_, whereas hypoxia (1% O_2_) was reached by incubation in a CO_2_/O_2_ incubator (Innova CO-14 incubator, New Brunswick Scientific CO, Edison, NJ).

### Flow cytometry and antibodies

To isolate LT-HSCs, ST-HSCs, MPPs, and LSK cells, mononuclear cells from BM were incubated with CD16/CD32 (Fc)-block (2.4G2, BD Biosciences, San Jose, CA). For isolation of LSK cells, APC- (BD Biosciences) or FITC-conjugated CD117 (2B8) and FITC- or Pacific Blue^™^-conjugated Sca-1 (D7) antibodies were added. For isolation of LT-HSC (CD34^-^FLT3^-^), ST-HSC (CD34^+^FLT3^-^), and MPP (CD34^+^FLT3^+^) cells, FLT3-Biotin (A2F10, eBioscience, San Diego, CA), streptavidin-Pe-Cy7 (eBioscience) and CD34-PE (MEC14.7) antibodies were added. Pe-Cy5-conjugated antibodies were used against lineage markers Gr-1 (RB6-8C5), Mac-1 (M1/70), B220 (RA-3-6B2), CD3 (145-2c11) and Ter-119. All antibodies were purchased from BioLegend (San Diego, CA) unless stated otherwise. Dead cells were detected with propidium iodide (1μg/mL, Molecular Probes, Eugene, OR) or 7-AAD (0.5μg/mL, Sigma Aldrich). Labeled cells were analyzed on FACSCanto^™^ II, or sorted on a FACSAria^™^ (both BD Biosciences). Re-analysis of sorted cells showed purity above 96%.

### Methylcellulose colonies

MethoCult^®^ methylcellulose medium with recombinant cytokines (StemCell Technologies) were diluted 1:10 with IMDM (PAA Laboratories) supplemented with 2% heat-inactivated FBS. 1 x 10^4^ unfractionated BM cells from pIpC–treated *Hif-1α*^*Δ/Δ*^ or *Hif-1α*^*+/+*^ mice were cultured in duplicate 35 mm dishes in normoxia. Colonies, mostly CFU-GM, were picked and cultured in IMDM supplemented with 20% heat-inactivated FBS, 50ng/mL SCF, and 25ng/mL murine IL-3 (both from Peprotech) for 48 hours at normoxia or hypoxia, after which the cells were lysed for PCR to evaluate successful gene ablation and qRT-PCR for gene expression analysis. For MPP colony formation, 500–1,000 GFP^+^ MPPs transduced with either one of two different Pdk1 shRNA lentivirus, or scramble control, were sorted into MethoCult^®^ methylcellulose medium with IMDM, 2% FBS, GM-CSF (5 ng/ml), IL-3 (10 ng/ml), SCF (50 ng/ml), and TPO (50 ng/ml) (all from Peprotech) and allowed to form colonies for 8 days.

### Lactate and ATP assays

Assays were performed using Lactate Assay kit and ATP bioluminescent assay kit (both from Sigma Aldrich) as recommended by the manufacturer. For lactate assays, approximately 5 x 10^4^ cells were harvested and homogenized in 4 volumes of the supplied lactate assay buffer before addition of supplied master reaction mix and measurement of absorption at 579 nm with a VERSA_max_ tunable microplate reader (Molecular Devices, Sunnyvale, CA). The lactate production was calculated from a standard curve. For ATP assays, 500 freshly isolated or cultured LSK cells were assayed for intracellular ATP level in a chemoluminometer (BioOrbit, Turku, Finland) with an appropriate ATP standard.

### Quantitative real-time PCR

Total RNA was isolated from LSK cells using RNeasy^®^ micro kit (Qiagen), DNase treatment included. For LT-HSCs, ST-HSCs, MPPs, c-kit^+^, or c-kit^-^, 6.500 to 20.000 cells were directly sorted into the buffer RLT contained in the RNeasy^®^ micro kit, and total RNA was extracted. cDNA was generated by annealing total RNA to random primers (60 min at 50°C and 15 min at 70°C) in reaction mixture containing 240 ng of random primer, 0.5 mM dNTP (Fermentas, Lithuania), 1x first strand buffer, 5 μM DTT, 2 units/μL RNaseOUT (Fermentas) and 5 units/μL SuperscriptIII reverse transcriptase (all reagents from Invitrogen unless stated otherwise). To quantify transcripts reactions were performed in 10 μL with 2x SYBR green master mix (Roche), 0.5 μM of forward and reverse primers, and 4–12 ng template, or FastStart Universal Probe Master (Rox) (Roche), 20x Assays-on-Demand^™^ probes (Applied Biosystems), RNase-free H_2_O and template equivalent to 370–2300 cells (freshly sorted) or 4-12ng template (cultured cells). Primers used are listed in S2 Table and probes in S3 Table. β-actin and Hprt were used for sample normalization. All samples were set in triplicates, non-template controls were used for all samples. qRT-PCR was initiated by holding for 10 min at 95°C and 40 cycles of 15s at 95°C and 60s at 60°C, and performed using 7900 HT Fast Real-time PCR System (Applied Biosystems). Relative expression was calculated by normalization to the reference gene.

### Lentiviral and retroviral vectors

Complementary DNA short hairpin (sh) RNA MISSION^™^ interference lentiviral vectors targeting Pdk1 as well as non-targeting shRNA scramble control were designed by the RNAi Consortium (TRC) at the Broad institute and purchased from Sigma (listed in S4 Table). The shRNAs were subcloned to the lentiviral vector pLKO.1-EGFP (kindly provided by Dr. J. Larsson, Lund). cDNA encoding murine *Hif-1α* carrying two point mutations rendering the protein insensitive to oxygen dependent degradation (*caHif-1α*; double mutant P402A/P563A) was cloned in the retroviral vector pMy-EGFP (kindly provided by Jörg Cammenga, Linköping). Virus supernatants were obtained by calcium phosphate transfection of 293T cells together with the helper plasmids pMD.G and pCMVΔR8.2 (lentivirus) or pVSV-G and pGAG-pol (retrovirus) as previously described [23]. Cells were transduced using Retronectin^™^ (Takara, Tokyo) and spinfection at 1,800g in the presence of 5 μg Polybrene/mL (Sigma Aldrich). GFP^+^ cells were sorted on a FACSAria^™^ (BD Biosciences).

### In vivo reconstitution

B6.SJL (CD45.1) and C57BL/6J (CD45.2) mice were used as donors and recipients, respectively. LT-HSCs, MPPs or LSK cells from CD45.1 mice were transduced with either one of two different *Pdk1* shRNA lentivirus, or scramble control. After 2 days, the percent GFP^+^ cells were assessed by flow cytometry and 7 x 10^4^ GFP^+^ LSK cells or MPPs, or 4.3 x 10^3^ GFP^+^ LT-HSCs, were injected in the lateral tail vein of lethally irradiated (9 Gy) CD45.2 mice along with 2 x 10^5^ BM supporter cells. Peripheral blood was collected by lateral tail vein bleeding of transplanted mice and stained with anti-CD45.1-Brilliant Violet 421 (A20), anti-CD45.2-PE/Cy7 (104–2), CD19-APC, B220-PE, Gr-1-APC-Cy7 and Mac-1-APC-Cy7 after red blood cell lysis using ammonium chloride. All antibodies were purchased from BioLegend. Dead cells were detected with 7-AAD (0.5μg/mL).

### Statistical analysis

Data are expressed as the mean + standard deviation (SD), median ± interquartile range, or median and ranges. Statistical analysis was performed using Student`s t-test and Mann-Whitney *U*-test by GraphPad Prism 7 (GraphPad Software Inc., La Jolla CA).

## Results

### Hypoxia upregulates PDK1 and converts energy metabolism of LSK mouse bone marrow cells from mitochondrial oxidative phosphorylation to glycolysis

**Fig 1:**
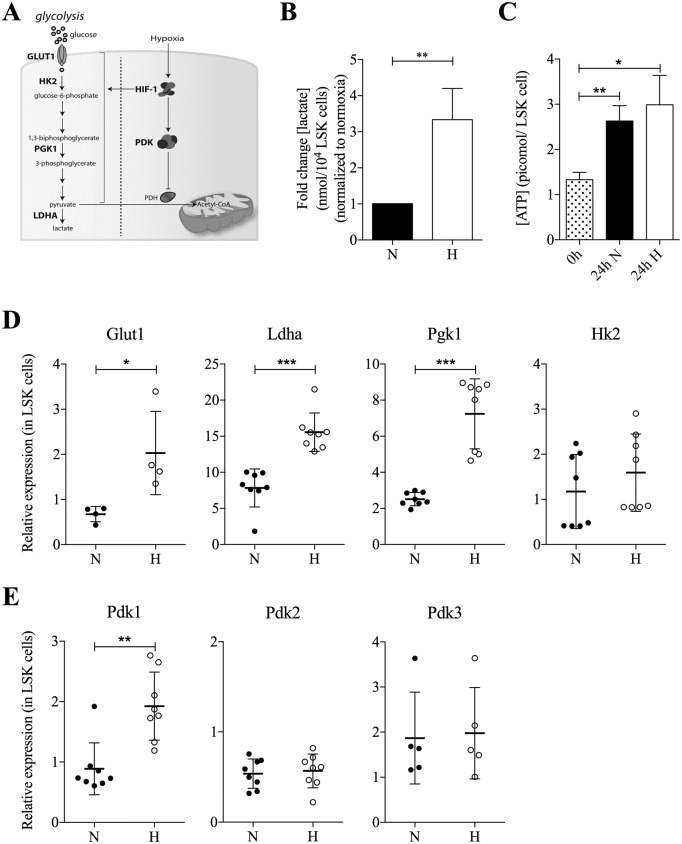
Hypoxia upregulates *Pdk1* expression and induces a metabolic shift to glycolysis in LSK cells from mouse bone marrow. (**A**) Hypoxia promotes glycolysis and *Pdk* activation. PDK subsequently prevents the conversion of pyruvate to acetyl-CoA by inhibiting pyruvate dehydrogenase (PDH). (**B**) Lactate production in the nmolar range in LSK cells from mouse bone marrow cultured for 48 hours in normoxia or hypoxia per 10,000 cells. Data in hypoxia were normalized to the values in normoxia and presented as fold change (n = 3, mean ± SD). Statistical analysis was performed using a student’s t-test. (**C**) Intracellular ATP concentration in LSK cells either freshly sorted or cultured for 24 hours in normoxia or hypoxia before sorting of viable cells. Data are presented as mean ± SD (n = 3). Statistical analysis was performed using a student’s t-test. (**D**) qRT-PCR analysis of *Glut1*, *Ldha*, *Pgk1*, and *Hk2* expression 24 hours after culture of LSK cells in normoxia or hypoxia. Data were normalized to *Hprt* expression (n = 4–8, in triplicates) and did not vary in expression between hypoxia and normoxia in LSK cells. Each dot represents one sample, and data are presented as median (horizontal line) ± interquartile range. Statistical analysis was performed using a Mann-Whitney *U* test. (**E**) *Pdk1-3* expression in LSK cells after 24 hour culture in normoxia or hypoxia. Data were normalized to *β-actin* x 10^−3^ expression and collected from eight (*Pdk1/2*) or five (*Pdk3*) individual experiments analyzed in triplicates. *Pdk4* was undetectable (S1 Fig). Each dot represents one sample, and data are presented as median (horizontal line) ± interquartile range. Statistical analysis was performed using a Mann-Whitney *U* test.

Measurements of the oxygen levels in the BM of living mice has displayed oxygen tension to be rather low and in the range of 10–30 mmHg in HSC niches [5]. This corresponds to an oxygen level of approximately 1–1.5% O_2_ and has been used by others and us in previous studies [23–26]. We first wanted to confirm that hypoxic exposure led to upregulation of genes associated to glycolytic activity (depicted schematic in Fig 1A). Twenty-four hours of incubation at 1% O_2_ of LSK cells led to a clear increase of lactate production (Fig 1B) and a slight increase of intracellular ATP production (Fig 1C). The RNA expression of glucose transporter *Glut1*, lactate dehydrogenase A (*Ldha*) and phosphoglycerate kinase 1 (*Pgk1*) increased, and a trend of higher expression of hexokinase 2 (*Hk2*) was observed (Fig 1D). *Pdk1* was also upregulated; however, hypoxia did not have any major effect on *Pdk2* and *Pdk3* (Fig 1E), and *Pdk4* expression was below the detection limit (S1 Fig).

It has previously been suggested that *Pdk2* and *Pdk4* expression is higher in more primitive progenitor cells [21]. We therefore analyzed whether *Pdk1* is distinctly expressed in various BM cell populations. We performed qPCR expression analysis of the *Pdk* gene family in LT-HSCs, ST-HSCs, and MPPs that are all included within LSK cells. It has been demonstrated that these progenitor populations form a sequential developmental lineage where LT-HSCs give rise to ST-HSCs followed by MPPs [27–29]. LT-HSCs have life-long self-renewal ability and contribute to long-term multi-lineage reconstitution of irradiated hosts upon transplantation. In contrast, ST-HSCs have limited self-renewal ability and can only support reconstitution of the hematopoietic system for about 6 weeks. MPPs can support the generation of all mature blood cells types but maintain no obvious self-renewal capacity, and as a consequence they can only support hematopoiesis transiently [28–30].

**Fig 2:**
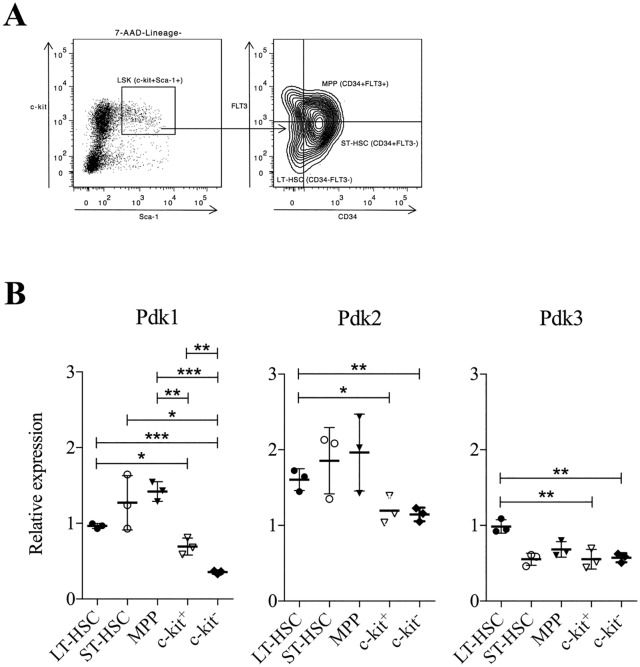
Primitive HSCs express higher levels of *Pdk1* compared to more differentiated cells. (**A**) Example dot plots of flow cytometry gating strategy for LSK cells, LT-HSCs (CD34^-^FLT3^-^), ST-HSCs (CD34^+^FLT3^-^), and MPPs (CD34^+^FLT3^+^). 7AAD^-^Lineage^-^ cells were positively selected for Sca-1 and c-kit to sort out LSK cells. (**B**) *Pdk1-3* expression in sorted LT-HSCs, ST-HSCs, MPPs cells, and differentiated c-kit^+^ or c-kit^-^ cells by qRT-PCR. Data were normalized to *Hprt* expression (n = 3, in triplicates). Each dot represents one sample, and data are presented as mean (horizontal line) ± SD. Statistical analysis was performed using a student’s t-test. N: normoxia, H: hypoxia. *, *P* < .05; **, *P* < .01; ***, *P* < .001.

Fig 2A shows the gating strategy by FACS for isolating the three cell populations from the BM. We also included progenitor cells expressing or lacking c-kit expression as two examples of more differentiated progeny where c-kit^-^ cells are the most differentiated. Expression analysis demonstrated that LT-HSCs, ST-HSCs, and MPPs expressed higher levels of *Pdk1* compared to more differentiated c-kit^+^ and c-kit^-^ cells (Fig 2B). In addition, expression of *Pdk2* and *Pdk3* was higher in LT-HSCs compared to differentiated cells (Fig 2B), whereas *Pdk4* was not detected (not shown).

### *Pdk1* expression is upregulated by HIF-1α during hypoxic culture of LSK cells *in vitro*, but *Pdk1* is not an HIF-1α target *in vivo*

**Fig 3:**
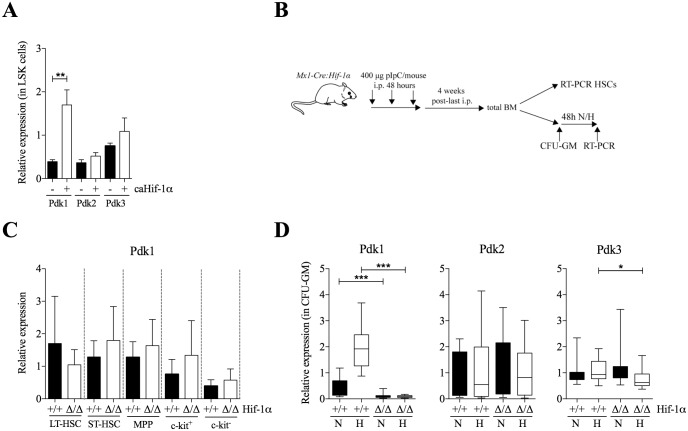
*Pdk1* is a major HIF-1 target in HSCs. (**A**) *Pdk1-4* expression in LSK cells transduced with *caHif-1α* or with empty vector, 24 hours after culture in normoxia. LSK cells were sorted for GFP expression 2 days after transduction. Data were normalized to *β-actin* x 10^−3^ expression (n = 3, in triplicates). Data are presented as mean (horizontal line) ± SD. Statistical analysis was performed using a student’s t-test. *Pdk4* expression was under the detection limit (not shown). (**B**) Experimental design of conditional gene targeting by pIpC-induced knockout in control or *Mx1-Cre*:*Hif-1α*^*flox/flox*^ mice. (**C**) qRT-PCR analysis of *Pdk1* RNA expression *in vivo* in FACS-sorted LT-HSCs, ST-HSCs, MPPs, and differentiated c-kit^+^ or c-kit^-^ cells from BM of *Hif-1α*^*+/+*^ or *Hif-1α*^*Δ/Δ*^ mice. Data were normalized to the expression of *Hprt* and collected from three individual experiments analyzed in triplicate qRT-PCRs. Data are presented as mean ± SD. Statistical analysis was performed using a student’s t-test. (**D**) qRT-PCR analysis of *Pdk1-4* expression in *Hif-1α*^*+/+*^ or *Hif-1α*^*Δ/Δ*^ in colony forming unit-granulocyte/monocyte (CFU-GM) after 10 days in methylcellulose and normoxia before 48 hours in normoxia or hypoxia. Data were normalized to *Hprt* expression and collected from 16 (*Hif-1α*^*+/+*^) or 14 (*Hif-1α*^*Δ/Δ*^) individual colonies analyzed in triplicates by qRT-PCR. Data represent median and ranges. Statistical analysis was performed using a Mann-Whitney *U* test. *Pdk4* expression was undetected (not shown). N: normoxia, H: hypoxia. *, *P* < .05; **, *P* < .01; ***, *P* < .001.

To investigate if the upregulation of *Pdk1* in hypoxia was mediated by HIF-1α, LSK cells were transduced with constitutively active and oxygen-insensitive *Hif-1α* (*caHif-1α*), previously shown by us to be stable at the protein level in ambient air [23]. In normoxia, overexpression of *caHif-1α* led to increased *Pdk1* expression but not of *Pdk2* or *Pdk3* (Fig 3A). To define the *in vivo* role of HIF-1α for *Pdk1* expression, we deleted *Hif-1α* specifically in hematopoietic cells using *Mx1-Cre*:*Hif1α*^*flox/flox*^ (*Hif-1α*^*Δ/Δ*^) mice by multiple pIpC-injections. Four weeks after treatment, the effect of HIF-1α deficiency was assessed by quantitative real-time (qRT-PCR) analysis in sorted BM populations (experimental design in Fig 3B). The efficiency of *Hif-1α* ablation was assessed by PCR analysis of genomic DNA in total BM, showing nearly complete knockout (S2A Fig). Although there was a trend towards decreased expression of *Pdk1* in LT-HSCs from *Hif-1α*^*Δ/Δ*^ mice compared to ST-HSCs, MPPs, c-kit^+^ and c-kit^-^ cells, the difference was not significant (Fig 3C).

To directly measure the effects of hypoxia on progenitors lacking HIF-1α, we decided to determine the expression of *Pdk1* in BM progenitors isolated from *Hif-1α*^*Δ/Δ*^ mice upon exposure to hypoxia *in vitro*. The analysis was performed by seeding BM cells from either *Hif-1α*^*+/+*^ or *Hif-1α*^*Δ/Δ*^ mice in semi-solid methylcellulose, and allowing colonies to form in normoxia for 9–12 days. Then multiple individual colonies (mainly colony forming unit-granulocyte/macrophage; CFU-GM) were isolated and examined for deletion of *Hif-1α* by PCR analysis (S2B Fig). The remaining cells from each colony were grown in suspension for an additional 48 hours in normoxia or hypoxia, after which RNA was isolated and expression of *Pdk1-4* was determined. In hypoxia, expression of *Pdk1* was reduced 22-fold and almost completely abolished in colonies of *Hif-1α*^*Δ/Δ*^ mice (Fig 3D). There was also a decrease in *Pdk3* expression whereas *Pdk2* was unaffected. This observation is consistent with a role of HIF-1α in regulating *Pdk1* expression. However, in normoxia deletion of *Hif-1α* also led to decreased expression of *Pdk1*, although at a lower level (3.8-fold). These findings suggest that *Pdk1* is a target for HIF-1α, but that HIF-1α can regulate *Pdk1* even in a non-hypoxic environment. Since no effects of *Pdk1* expression was seen in BM cells of *Hif-1α*^*Δ/Δ*^ mice, the results raise questions to whether HIF-1α regulates *Pdk1* differently during *in vitro* and *in vivo* conditions.

### PDK1 is important for long-term hematopoietic engraftment

**Fig 4:**
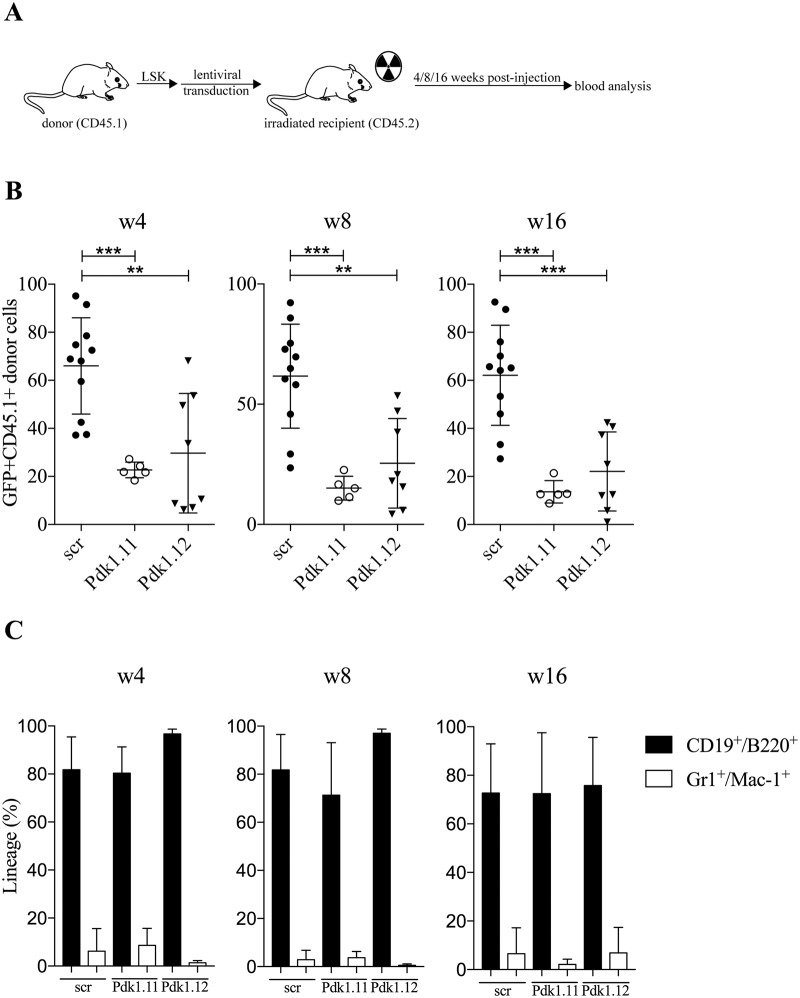
PDK1 is essential for hematopoietic reconstitution. (**A**) Experimental design of mice transplanted with LSK cells with silenced *Pdk1* expression using two different shRNA lentiviruses. (**B**-**C)** CD45.2 mice were transplanted with 7 x 10^4^ GFP^+^ LSK cells from CD45.1 mice transduced with non-targeting shRNA-scramble control (n = 11) or two different shRNA-*Pdk1* (n = 14) along with 2 x 10^5^ BM supporter cells. Total percent of GFP^+^ donor cells (**B**) or myeloid (Gr1^+^Mac-1^+^) and lymphoid (CD19^+^B220^+^) cells (**C**) in peripheral blood after transplantation was determined by flow cytometry after 4, 8 and 16 weeks. Each dot represents one mouse, and the data are presented as mean (horizontal line). Statistical analysis was performed using Mann-Whitney *U* test. **, *P* < .01; ***, *P* < .001.

Despite any clear evidence for HIF-1α-mediated regulation of *Pdk1 in vivo* utilizing *Hif-1α* ^*Δ/Δ*^ mice, we decided to assess the importance of PDK1 in transplantation assays to compare the ability of LSK cells with no PDK1 expression to reconstitute the hematopoietic system in irradiated host mice. This was done with LSK cells with silenced *Pdk1* expression using two different shRNA lentiviruses, both efficiently reducing the RNA level of *Pdk1* in both normoxia and hypoxia (S3 Fig). A non-targeting scramble shRNA was used as control. Forty-eight hour post-transduction, 7 x 10^4^ GFP^+^ LSK cells from CD45.1 mice were transplanted to CD45.2 mice along with BM supporter cells. Peripheral blood was analyzed for GFP expression after 4, 8, and 16 weeks (experiment depicted in Fig 4A). At all time, engraftment was significant lower in recipients transplanted with any of the two shRNAs to *Pdk1* compared to controls (Fig 4B). When the effects on myeloid and lymphoid contribution of transplanted cells were analyzed, no difference was seen between cells with control or *Pdk1* shRNA and both groups showed a clear bias towards lymphoid reconstitution (Fig 4C). This is in agreement with published results that engrafting LSK cells give mainly rise to lymphocytes in irradiated mice due to selective expansion of lymphoid progenitors [29, 31]. Taken together, the results demonstrate an important role of PDK1 during engraftment of HSCs to the BM of recipient mice but no distinct effect on any certain hematopoietic cell lineage.

### *Pdk1* silencing impairs MPP function both *in vitro* and *in vivo*

**Fig 5:**
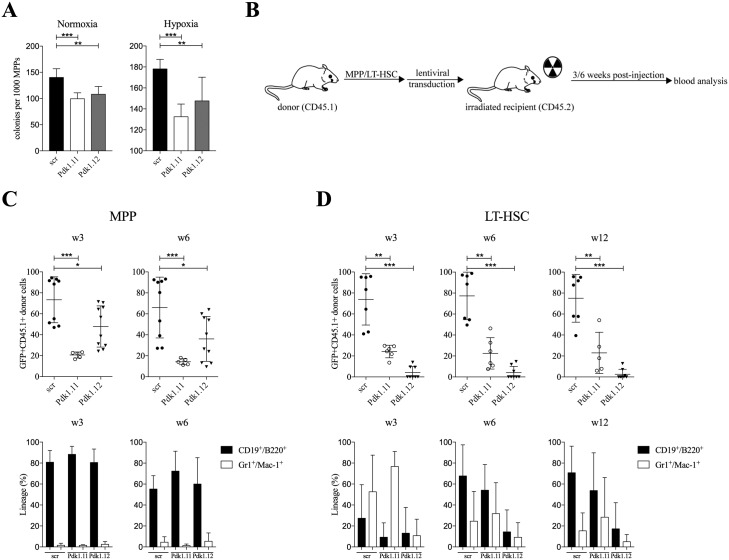
PDK1 is essential for both long- and short-term hematopoietic reconstitution. (**A**) MPPs silenced for *Pdk1* were plated after cell sorting in semi-solid methylcellulose media supplemented with cytokines and incubated in parallel plates either in hypoxia and normoxia. After 8 days the numbers of colonies were scored. Data are presented as mean ± SD (n = 6–8). Statistical analysis was performed using a student`s t-test. (**B**) Experimental design of mice transplanted with MPPs or LT-HSCs with silenced *Pdk1* expression using two different shRNA lentiviruses. (**C-D**) Freshly isolated MPPs and LT-HSCs from CD45.1 mice were transduced with shRNA-scramble control (n = 11) or two shRNAs to *Pdk1*. 7 x 10^4^ GFP^+^ MPP cells (**C**) or 4.3 x 10^3^ GFP^+^ LT-HSCs (**D**) were transplanted into CD45.2 mice along with 2 x 10^5^ BM supporter cells (n = 5-10/group). Total percent of GFP^+^ donor cells (upper panels) or the distribution between myeloid (Gr1^+^Mac-1^+^) versus lymphoid (CD19^+^B220^+^) cells (lower panels) in peripheral blood after transplantation was determined by flow cytometry after 3 and 6 weeks. Each dot represents one mouse, and the data are presented as mean (horizontal line). Statistical analysis was performed using Mann-Whitney *U* test. scr: scramble control. *, *P* < .05; **, *P* < .01; ***, *P* < .001.

Since our results implied that PDK1 was essential for HSC engraftment, and *Pdk1* expression was higher in both LT-HSCs and MPPs compared to later progeny, we decided to determine the effects of *Pdk1* silencing of MPPs *in vitro* and *in vivo*. We first tested if *Pdk1* silencing affected the clonality of MPPs by plating them (500–1,000 cells per plate) after cell sorting in semi-solid methylcellulose media supplemented with cytokines (GM-CSF, IL-3, SCF, and TPO). To determine if any effects were linked to hypoxia, MPPs were incubated in parallel plates either in hypoxia and normoxia. After 8 days the numbers of colonies were scored. Overall the plating efficiency was slightly higher in hypoxia. MPPs infected with either of the two *Pdk1* shRNAs displayed lower clonogenic potential in both conditions and the numbers of colonies decreased by approximately 25% (Fig 5A). This *in vitro* experiment indicates that although MPPs retain clonogenicity in the absence of PDK1, the ability is partially lost even in normoxia, and is thus not entirely dependent on hypoxia.

We then addressed whether PDK1 is important for short-term engraftment by transplantation of MPPs to recipient mice. To compare the effects of MPPs to another transplantable cell population, the time kinetics for engraftment of MPPs at 3 and 6 weeks were compared to LT-HSCs in parallel. The two cell populations were FACS-sorted and transplanted separately to recipient mice after *Pdk1* gene silencing. After 3 and 6 weeks, the percent GFP^+^ cells in peripheral blood was determined in mice (experiment depicted in Fig 5B) receiving either MPPs (Fig 5C) or LT-HCSs (Fig 5D). In both cases, there was a reduction in the engraftment potential of MPPs and LT-HSCs when expressing shRNA to *Pdk1* (Fig 5C and 5D). While the engraftment in mice transplanted with LT-HSCs was more strongly affected, the relative contribution of donor cells remained higher for MPPs. The reason for different effects by the two shRNAs to *Pdk1* on LT-HSCs and MPP, respectively, is not known but could be due to different levels of knockdown and dose-dependent effects. When the contribution between myeloid and lymphoid cells were analyzed after 3 and 6 weeks, there was no effect of *Pdk1* shRNA on lymphoid and myeloid cells in MPPs (Fig 5C). In LT-HSCs expressing shRNA to *Pdk1* there was a trend towards reduced lymphoid contribution but this was not statistically significant (Fig 5D). Taken together, the *in vivo* analyses corroborate on the essential role of PDK1 in both long- and short-term HSC populations.

## Discussion

In this study, we demonstrate that *Pdk1* is a HIF-1α-target gene in LSK cells *in vitro*, that *ex vivo* expanded BM colonies of *Hif-1α*^*Δ/Δ*^ mice cultured in hypoxia are unable to express *Pdk1*, and that PDK1 is essential for the integrity of both LT-HSCs and MPPs upon transplantation. Taken together, the results indicate that *Pdk1* is a hypoxia-inducible gene mediated by HIF-1α, and that PDK1 is involved in HSC function. This would be in agreement with a previous report that PDK1 expression is higher at the protein level in LSK cells compared with more differentiated cells [32]. In addition, we could demonstrate that LT-HSCs, ST-HSCs and MPPs expressed higher levels of *Pdk1* compared to more differentiated c-kit^+^ cells. While this was also the case for expression of *Pdk2* and *Pdk3*, expression of *Pdk4* was below the level of detection in our assay. However, we could not find evidence for any effects of conditional *Hif-1α* deficiency for the expression of *Pdk1*, or any of the other *Pdk* family genes, in different cells within the HSC compartment.

These results stands in contrast to what was reported by Takubo et al [21], in which they showed that *Pdk2* and *Pdk4* were decreased in LT-HSCs in *Hif-1α*^*Δ/Δ*^ mice. Similar to our results however, Takubo et al showed that *Pdk1* expression was higher in HSCs and early progenitors compared to later progeny. One plausible explanation to the discrepancies between the two studies could be the use of different mouse strains or the experimental design to induce conditional knockdown, which may lead to distinct effects in the BM. Moreover, the effects of gene ablation at steady state-hematopoiesis could be different than shRNA knockdown in transplantable BM cells. Since compensatory upregulation of HIF-2α in HSCs ablated for HIF-1α has been reported previously [33], this could also differ between the two studies.

Our results that PDK1 is essential for both LT-HSCs and transient MPPs imply that PDK1 has a broad function in multiple progenitor cells. This is supported by the *in vitro* clonogenic assay with MPPs where *Pdk1* silencing partial impaired colony formation. Although our study suggests that *Pdk1* is a target for HIF-1α in HSCs, *Pdk1* expression was also reduced in colonies with deleted *Hif-1α* grown in normoxia. This indicates that *Pdk1* can be controlled by both hypoxia-dependent and -independent mechanisms, both however requiring HIF-1α activity. Previous studies have demonstrated that SCF and TPO with positive effects on HSCs can activate HIF-1α under normoxic conditions [34, 35]. In addition, HIF-1α contributes to regulation of growth factor-stimulated metabolism in the absence of hypoxia [36]. Since both SCF and TPO were included in our cultures, the results corroborate on an essential role of PDK1 in both long- and short-term HSC populations, but that PDK1 expression is not exclusively dependent on hypoxia.

Concordant it was recently shown that HSCs from *Arnt*^*Δ/Δ*^ mice deficient in the common β subunit of Hif (*Hif-1α*) or double *Hif-1α*^*Δ/Δ*^ x *Hif-2α*^*Δ/Δ*^ knockout mice have very small effects on the expression of *Pdk1*, *Pdk2* and *Pdk4* [14]. In addition, a recent report has shown that inducible deletion of *Hif-1α* had no impact on HSC survival [15]. Thus, although *Pdk* family members are expressed at high levels in HSCs, and PDK1 appears important for proper HSCs function upon transplantation to recipient mice, the link between HIF regulation on *Pdk* activity is still an open issue.

Recently, it was demonstrated that HSCs upon transplantation do not seem to seek out specific hypoxic niches as sites for preferential homing in post-chemotherapy or radiation-ablated BM [5]. Since the oxygen tension is elevated in ablated BM [5], it is possible that the effect of silencing of *Hif-1α* or any of the *Pdk* genes could differ between HSC transplantation and at steady state hematopoiesis. If the hypoxic status of HSCs and more committed progenitors is related to cell-specific mechanisms rather than different oxygen levels at any defined regions [37], LT-HSCs, ST-HSCs, and MPPs could all be located in hypoxic BM regions and depend on PDK1 for proper function. Given that our study indicates *Pdk1* expression at equal levels in LT-HSCs and MPPs, but at higher levels compared to differentiated c-kit^-^ cells, PDK1 appears to be important for both HSCs and multipotent progenitor cells.

## Conclusions

Our study demonstrates for the first time that PDK1 is a critical factor for engraftment of HSCs and multipotent progenitors. The results that both long-term and short-term reconstitution was impaired when *Pdk1* expression was silenced suggest that PDK1 is essential not only in LT-HSCs but also in multipotent progenitor cells. Therefore, we believe that PDK1 plays a significant role in hematopoiesis at transplantation, but that it also has broad effects in multiple stem and progenitor cells. The exact role of each individual PDK member needs to be further explored to determine if they have distinct, similar and/or overlapping function. Due to conflicting results to the importance of HIF-1α to maintain HSCs, it also remains to clarify if PDK1 function in the BM is dependent on its hypoxic nature and whether *Pdk1*, and other *Pdk* genes, are under HIF-1α control.

## Supporting information

S1 Fig Gene expression analysis of *Pdk4* in muscle, liver, and LSK cells from mouse bone marrow. qRT-PCR analysis was performed and data were normalized to *Hprt* expression (n = 2–4, in triplicates). Each dot represents one sample, and the data are presented as mean (horizontal line) ± SD. nd, not detected. *Pdk4* was undetectable in LSK cells.
(TIFF)

S2 Fig *Hif-1α* knockout efficiency analyzed by genotyping. Genotype analysis of unfractionated BM cells from pIpC–treated *Hif-1α*^*+/+*^ and *Hif-1α*^*Δ/Δ*^ mice (A) or CFU-GM colonies from one representative *Hif-1α*^*Δ/Δ*^ mouse (B). The deleted exon 2 of the *Hif-1α* gene is indicated by the arrow (300bp).
(TIFF)

S3 Fig Efficient gene silencing with shRNA to Pdk1. LSK cells were transduced with two different shRNAs to Pdk1 or scramble shRNA as control. Forty-eight hours after transduction, cells were sorted for GFP expression and then incubated for 24 hours in hypoxia or normoxia after which qRT-PCR analysis was performed for expression of Pdk1. The data were normalized to the expression of β-actin (n = 3, in triplicates). Each dot represents the mean value of one sample (horizontal line) ± SD. Statistical analysis was performed using a student’s t-test. *, *P* < .05; **, *P* < .01.
(TIFF)

S1 Table Sequences of primers used in PCR amplification and sequencing reactions. (PDF)

S2 Table Sequences of primers used in qRT-PCR analysis. (PDF)

S3 Table Probes used in qRT-PCR analysis. (PDF)

S4 Table shRNA sequences used in the pLKO.1-GFP lentiviral vector. (PDF)

S5 Table Complete data set Figs 1–5. All data generated for results presented in Figs 1–5 are provided as supplementary file containing the raw daya from APT and lactate measurements as well as RT-PCR analyses, and raw data from the fcs-files collected by flow cytometry of transplantation experiments.
(DOCX)

## References

[1] Boulais PE, Frenette PS. Making sense of hematopoietic stem cell niches. Blood. 2015;125(17):2621–9. 10.1182/blood-2014-09-570192 25762174 PMC4408288

[2] Morrison SJ, Scadden DT. The bone marrow niche for haematopoietic stem cells. Nature. 2014;505(7483):327–34. 10.1038/nature12984 24429631 PMC4514480

[3] Morikawa T, Takubo K. Hypoxia regulates the hematopoietic stem cell niche. Pflugers Archiv: European journal of physiology. 2015; 10.1007/s00424-015-1743-z 26490456

[4] Parmar K, Mauch P, Vergilio JA, Sackstein R, Down JD. Distribution of hematopoietic stem cells in the bone marrow according to regional hypoxia. Proc Natl Acad Sci USA. 2007;104(13):5431–6. Epub 2007/03/22. 10.1073/pnas.0701152104 17374716 PMC1838452

[5] Spencer JA, Ferraro F, Roussakis E, Klein A, Wu J, Runnels JM, et al Direct measurement of local oxygen concentration in the bone marrow of live animals. Nature. 2014;508:269–73. Epub 10.1038/nature13034 24590072 PMC3984353

[6] Suda T, Takubo K, Semenza GL. Metabolic regulation of hematopoietic stem cells in the hypoxic niche. Cell Stem Cell. 2011;9(4):298–310. Epub 2011/10/11. 10.1016/j.stem.2011.09.010 21982230

[7] Ito K, Hirao A, Arai F, Takubo K, Matsuoka S, Miyamoto K, et al Reactive oxygen species act through p38 MAPK to limit the lifespan of hematopoietic stem cells. Nature medicine. 2006;12(4):446–51. 10.1038/nm1388 16565722

[8] Simsek T, Kocabas F, Zheng J, Deberardinis RJ, Mahmoud AI, Olson EN, et al The distinct metabolic profile of hematopoietic stem cells reflects their location in a hypoxic niche. Cell Stem Cell. 2010;7(3):380–90. Epub 2010/09/02. 10.1016/j.stem.2010.07.011 20804973 PMC4159713

[9] Kallio PJ, Pongratz I, Gradin K, McGuire J, Poellinger L. Activation of hypoxia-inducible factor 1alpha: posttranscriptional regulation and conformational change by recruitment of the Arnt transcription factor. Proc Natl Acad Sci USA. 1997;94(11):5667–72. Epub 1997/05/27. 9159130 10.1073/pnas.94.11.5667 PMC20836

[10] Wang GL, Jiang BH, Rue EA, Semenza GL. Hypoxia-inducible factor 1 is a basic-helix-loop-helix-PAS heterodimer regulated by cellular O2 tension. Proc Natl Acad Sci USA. 1995;92(12):5510–4. Epub 1995/06/06. 7539918 10.1073/pnas.92.12.5510 PMC41725

[11] Huang LE, Arany Z, Livingston DM, Bunn HF. Activation of hypoxia-inducible transcription factor depends primarily upon redox-sensitive stabilization of its alpha subunit. J Biol Chem. 1996;271(50):32253–9. Epub 1996/12/13. 8943284 10.1074/jbc.271.50.32253

[12] Nakayama K, Frew IJ, Hagensen M, Skals M, Habelhah H, Bhoumik A, et al Siah2 regulates stability of prolyl-hydroxylases, controls HIF1alpha abundance, and modulates physiological responses to hypoxia. Cell. 2004;117(7):941–52. Epub 2004/06/24. 10.1016/j.cell.2004.06.001 15210114

[13] Takubo K, Goda N, Yamada W, Iriuchishima H, Ikeda E, Kubota Y, et al Regulation of the HIF-1alpha level is essential for hematopoietic stem cells. Cell Stem Cell. 2010;7(3):391–402. Epub 2010/09/02. 10.1016/j.stem.2010.06.020 20804974

[14] Krock BL, Eisinger-Mathason TS, Giannoukos DN, Shay JE, Gohil M, Lee DS, et al The aryl hydrocarbon receptor nuclear translocator is an essential regulator of murine hematopoietic stem cell viability. Blood. 2015;125(21):3263–72. 10.1182/blood-2014-10-607267 25855602 PMC4440881

[15] Vukovic M, Sepulveda C, Subramani C, Guitart AV, Mohr J, Allen L, et al Adult hematopoietic stem cells lacking Hif-1alpha self-renew normally. Blood. 2016;127(23):2841–6. 10.1182/blood-2015-10-677138 27060169 PMC4956613

[16] Kim JW, Tchernyshyov I, Semenza GL, Dang CV. HIF-1-mediated expression of pyruvate dehydrogenase kinase: a metabolic switch required for cellular adaptation to hypoxia. Cell Metab. 2006;3(3):177–85. Epub 2006/03/07. 10.1016/j.cmet.2006.02.002 16517405

[17] Papandreou I, Cairns RA, Fontana L, Lim AL, Denko NC. HIF-1 mediates adaptation to hypoxia by actively downregulating mitochondrial oxygen consumption. Cell Metab. 2006;3(3):187–97. Epub 2006/03/07. 10.1016/j.cmet.2006.01.012 16517406

[18] Prigione A, Rohwer N, Hoffmann S, Mlody B, Drews K, Bukowiecki R, et al HIF1alpha Modulates Cell Fate Reprogramming Through Early Glycolytic Shift and Upregulation of PDK1-3 and PKM2. Stem Cells. 2014;32(2):364–76. Epub 2013/10/15. 10.1002/stem.1552 24123565 PMC5730046

[19] Gudi R, Bowker-Kinley MM, Kedishvili NY, Zhao Y, Popov KM. Diversity of the pyruvate dehydrogenase kinase gene family in humans. J Biol Chem. 1995;270(48):28989–94. Epub 1995/12/01. 7499431 10.1074/jbc.270.48.28989

[20] Rowles J, Scherer SW, Xi T, Majer M, Nickle DC, Rommens JM, et al Cloning and characterization of PDK4 on 7q21.3 encoding a fourth pyruvate dehydrogenase kinase isoenzyme in human. J Biol Chem. 1996;271(37):22376–82. Epub 1996/09/13. 8798399 10.1074/jbc.271.37.22376

[21] Takubo K, Nagamatsu G, Kobayashi CI, Nakamura-Ishizu A, Kobayashi H, Ikeda E, et al Regulation of glycolysis by Pdk functions as a metabolic checkpoint for cell cycle quiescence in hematopoietic stem cells. Cell Stem Cell. 2013;12(1):49–61. Epub 2013/01/08. 10.1016/j.stem.2012.10.011 23290136 PMC6592822

[22] Ryan HE, Poloni M, McNulty W, Elson D, Gassmann M, Arbeit JM, et al Hypoxia-inducible factor-1alpha is a positive factor in solid tumor growth. Cancer Res. 2000;60(15):4010–5. Epub 2000/08/17. 10945599

[23] Eliasson P, Rehn M, Hammar P, Larsson P, Sirenko O, Flippin LA, et al Hypoxia mediates low cell-cycle activity and increases the proportion of long-term-reconstituting hematopoietic stem cells during in vitro culture. Exp Hematol. 2010;38(4):301–10 e2. Epub 2010/02/09. 10.1016/j.exphem.2010.01.005 20138114

[24] Kubota Y, Takubo K, Suda T. Bone marrow long label-retaining cells reside in the sinusoidal hypoxic niche. Biochem Biophys Res Commun. 2008;366(2):335–9. Epub 2007/12/01. 10.1016/j.bbrc.2007.11.086 18047833

[25] Shima H, Takubo K, Iwasaki H, Yoshihara H, Gomei Y, Hosokawa K, et al Reconstitution activity of hypoxic cultured human cord blood CD34-positive cells in NOG mice. Biochem Biophys Res Commun. 2009;378(3):467–72. Epub 2008/11/27. 10.1016/j.bbrc.2008.11.056 19032938

[26] Tang Y, Halvarsson C, Eliasson P, Jonsson JI. Hypoxic and normoxic in vitro cultures maintain similar numbers of long-term reconstituting hematopoietic stem cells from mouse bone marrow. Exp Hematol. 2012;40(11):879–81. 10.1016/j.exphem.2012.07.005 22820086

[27] Adolfsson J, Mansson R, Buza-Vidas N, Hultquist A, Liuba K, Jensen CT, et al Identification of Flt3+ lympho-myeloid stem cells lacking erythro-megakaryocytic potential a revised road map for adult blood lineage commitment. Cell. 2005;121(2):295–306. 10.1016/j.cell.2005.02.013 15851035

[28] Christensen JL, Weissman IL. Flk-2 is a marker in hematopoietic stem cell differentiation: a simple method to isolate long-term stem cells. Proc Natl Acad Sci USA. 2001;98(25):14541–6. Epub 2001/11/29. 10.1073/pnas.261562798 11724967 PMC64718

[29] Yang L, Bryder D, Adolfsson J, Nygren J, Mansson R, Sigvardsson M, et al Identification of Lin(-)Sca1(+)kit(+)CD34(+)Flt3- short-term hematopoietic stem cells capable of rapidly reconstituting and rescuing myeloablated transplant recipients. Blood. 2005;105(7):2717–23. 10.1182/blood-2004-06-2159 15572596

[30] Pietras EM, Reynaud D, Kang YA, Carlin D, Calero-Nieto FJ, Leavitt AD, et al Functionally Distinct Subsets of Lineage-Biased Multipotent Progenitors Control Blood Production in Normal and Regenerative Conditions. Cell Stem Cell. 2015;17(1):35–46. 10.1016/j.stem.2015.05.003 26095048 PMC4542150

[31] Kondo M. Lymphoid and myeloid lineage commitment in multipotent hematopoietic progenitors. Immunol Rev. 2010;238(1):37–46. 10.1111/j.1600-065X.2010.00963.x 20969583 PMC2975965

[32] Klimmeck D, Hansson J, Raffel S, Vakhrushev SY, Trumpp A, Krijgsveld J. Proteomic cornerstones of hematopoietic stem cell differentiation: distinct signatures of multipotent progenitors and myeloid committed cells. Molecular & Cellular Proteomics. 2012;11(8):286–302. Epub 2012/03/29. 22454540 10.1074/mcp.M111.016790 PMC3412962

[33] Kocabas F, Zheng J, Thet S, Copeland NG, Jenkins NA, DeBerardinis RJ, et al Meis1 regulates the metabolic phenotype and oxidant defense of hematopoietic stem cells. Blood. 2012;120(25):4963–72. 10.1182/blood-2012-05-432260 22995899 PMC3525021

[34] Pedersen M, Lofstedt T, Sun J, Holmquist-Mengelbier L, Pahlman S, Ronnstrand L. Stem cell factor induces HIF-1alpha at normoxia in hematopoietic cells. Biochem Biophys Res Commun. 2008;377(1):98–103. Epub 2008/10/07. 10.1016/j.bbrc.2008.09.102 18834862

[35] Kirito K, Fox N, Komatsu N, Kaushansky K. Thrombopoietin enhances expression of vascular endothelial growth factor (VEGF) in primitive hematopoietic cells through induction of HIF-1alpha. Blood. 2005;105(11):4258–63. 10.1182/blood-2004-07-2712 15705785 PMC1895043

[36] Lum JJ, Bui T, Gruber M, Gordan JD, DeBerardinis RJ, Covello KL, et al The transcription factor HIF-1alpha plays a critical role in the growth factor-dependent regulation of both aerobic and anaerobic glycolysis. Genes Dev. 2007;21(9):1037–49. 10.1101/gad.1529107 17437992 PMC1855230

[37] Nombela-Arrieta C, Pivarnik G, Winkel B, Canty KJ, Harley B, Mahoney JE, et al Quantitative imaging of haematopoietic stem and progenitor cell localization and hypoxic status in the bone marrow microenvironment. Nat Cell Biol. 2013;15(5):533–43. Epub 2013/04/30. 10.1038/ncb2730 23624405 PMC4156024

