# Supplementary figures and images for: Pyruvate dehydrogenase kinase 1 is essential for transplantable mouse bone marrow hematopoietic stem cell and progenitor function
Source: PLoS One. 2017 Feb 9;12(2):e0171714. doi: 10.1371/journal.pone.0171714 (PMC5300157; doi:10.1371/journal.pone.0171714)

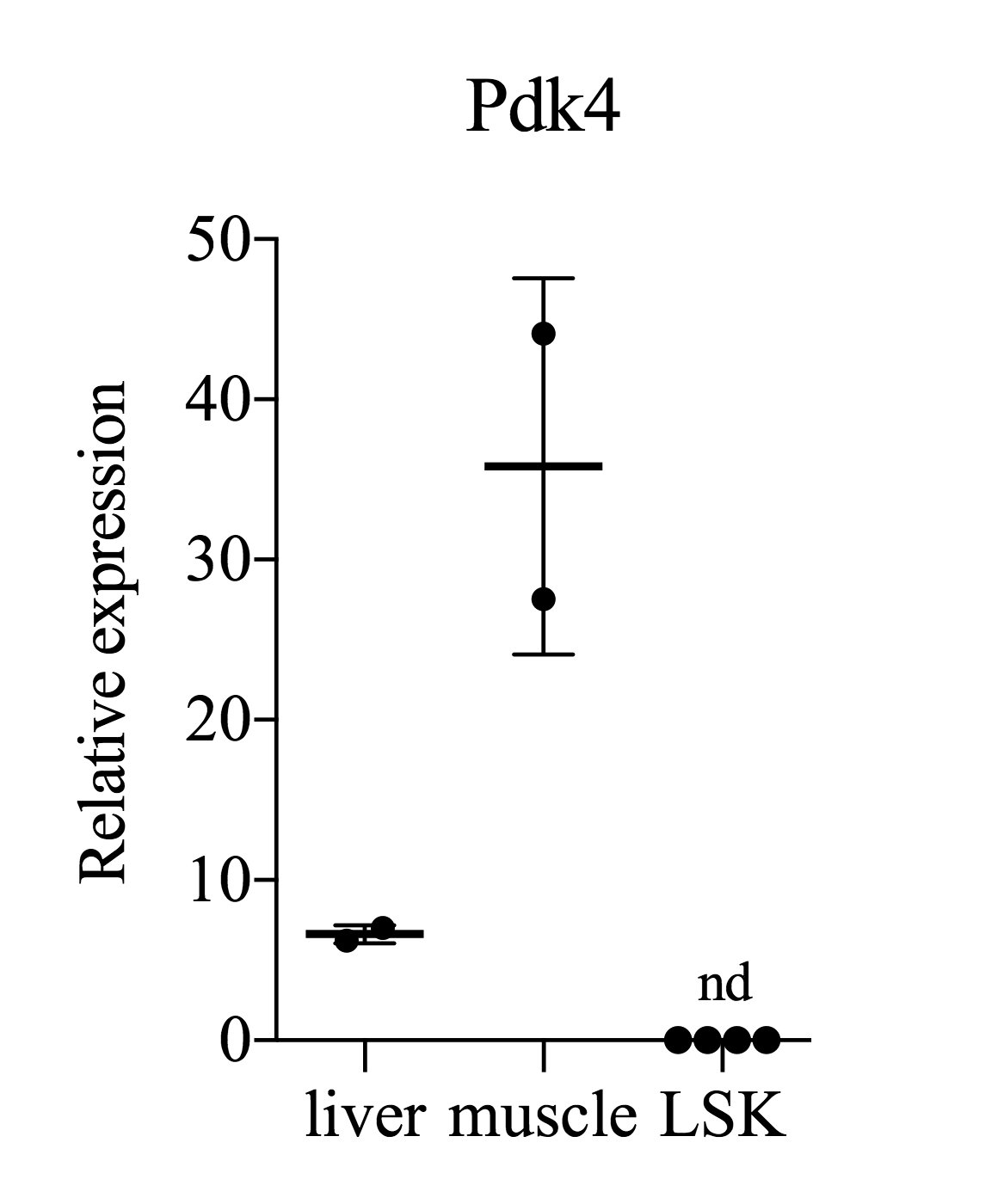

Supplement: S1 Fig — qRT-PCR analysis was performed and data were normalized to Hprt expression (n = 2–4, in triplicates). Each dot represents one sample, and the data are presented as mean (horizontal line) ± SD. nd, not detected. Pdk4 was undetectable in LSK cells. (TIFF) [file pone.0171714.s001.tiff]

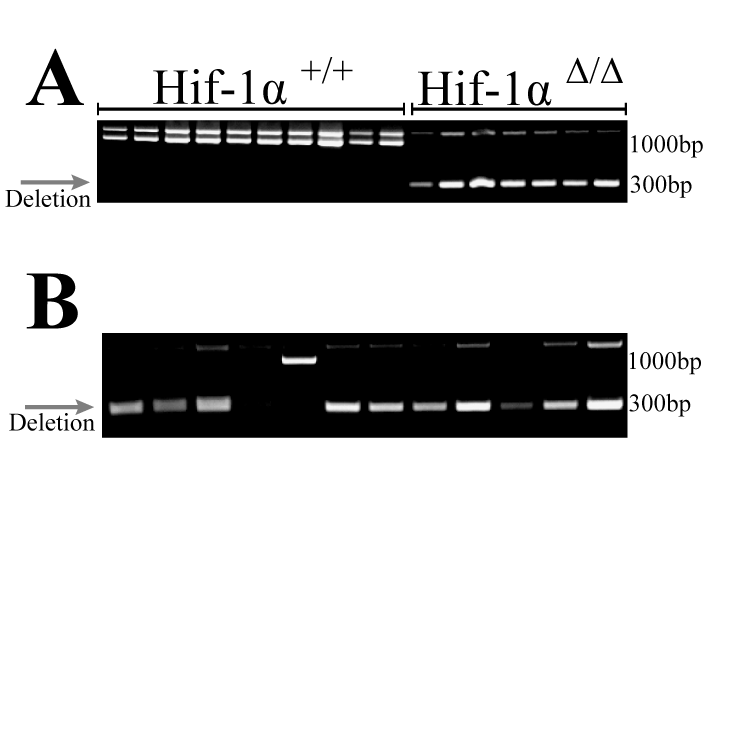

Supplement: S2 Fig — Genotype analysis of unfractionated BM cells from pIpC–treated Hif-1α+/+ and Hif-1αΔ/Δ mice (A) or CFU-GM colonies from one representative Hif-1αΔ/Δ mouse (B). The deleted exon 2 of the Hif-1α gene is indicated by the arrow (300bp). (TIFF) [file pone.0171714.s002.tiff]

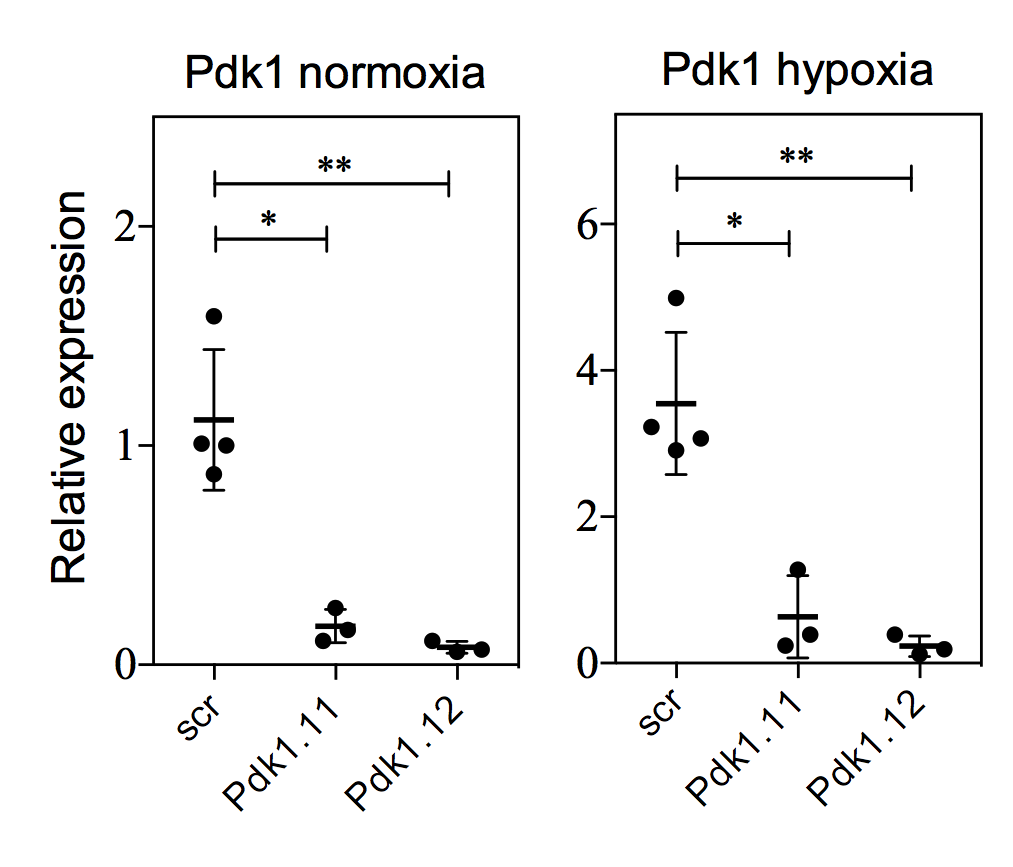

Supplement: S3 Fig — LSK cells were transduced with two different shRNAs to Pdk1 or scramble shRNA as control. Forty-eight hours after transduction, cells were sorted for GFP expression and then incubated for 24 hours in hypoxia or normoxia after which qRT-PCR analysis was performed for expression of Pdk1. The data were normalized to the expression of β-actin (n = 3, in triplicates). Each dot represents the mean value of one sample (horizontal line) ± SD. Statistical analysis was performed using a student’s t-test. *, P < .05; **, P < .01. (TIFF) [file pone.0171714.s003.tiff]
