# Supplementary material for: Pyruvate dehydrogenase kinase 1 is essential for transplantable mouse bone marrow hematopoietic stem cell and progenitor function
Source: PLoS One. 2017 Feb 9;12(2):e0171714. doi: 10.1371/journal.pone.0171714 (PMC5300157; doi:10.1371/journal.pone.0171714)
Supplement: S1 Table — (PDF) [file pone.0171714.s004.pdf]

Primer name	*Sequence 5'->3'*
Forward primer Mx1:Cre	CATGTGTCTTGGTGGGCTGAG
Reverse primer Mx1:Cre	CGCATAACCAGTGAAACAGCAT
Forward primer Hif-1α$^{flox/flox}$	CGTGTGAGAAAACTTCTGGATG
Reverse primer Hif-1α$^{flox/flox}$	AAAAGTATTGTGTTGGGGCAGT
Forward primer Hif-1α$^{\Delta/\Delta}$	GCAGTTAAGAGCACTAGTTG
Reverse primer Hif-1α$^{\Delta/\Delta}$	TTGGGGATGAAAACATCTGC
