# Supplementary material for: Pyruvate dehydrogenase kinase 1 is essential for transplantable mouse bone marrow hematopoietic stem cell and progenitor function
Source: PLoS One. 2017 Feb 9;12(2):e0171714. doi: 10.1371/journal.pone.0171714 (PMC5300157; doi:10.1371/journal.pone.0171714)
Supplement: S2 Table — (PDF) [file pone.0171714.s005.pdf]

Primer name	*Sequence 5'->3'*
Forward primer Hif-1α	TGACGGCGACAGGGTTTACA
Reverse primer Hif-1α	AATATGGCCCGTGCAGTGAA
Forward primer Pdk1	TCCCCCGATTCAGGTTCAC
Reverse primer Pdk1	CCCGGTCACTCATCTTCACA
Forward primer Pdk2	CACCGGACTCTAAGCCAGTT
Reverse primer Pdk2	ACGGGGTCATCTCCATAGGT
Forward primer Pdk3	GGGTACCGATGCAGTCATTTA
Reverse primer Pdk3	GCTTCAGGAGTGGTCTTGTAAT
Forward primer Glut1	TCACTGTGGTGTCGCTGTTT
Reverse primer Glut1	CCATGGAATAGGACCAGGGC
Forward primer β-actin	GCTGTATTCCCCTCCATCGTG
Reverse primer β-actin	CACGGTTGGCCTTAGGGTTCAG
Forward primer Hprt	GGCCAGACTTTGTTGGATTTG
Reverse primer Hprt	CGCTCATCTTAGGCTTTGTATTTG
