# Supplementary material for: Pyruvate dehydrogenase kinase 1 is essential for transplantable mouse bone marrow hematopoietic stem cell and progenitor function
Source: PLoS One. 2017 Feb 9;12(2):e0171714. doi: 10.1371/journal.pone.0171714 (PMC5300157; doi:10.1371/journal.pone.0171714)
Supplement: S3 Table — (PDF) [file pone.0171714.s006.pdf]

Probe name	*Assay ID*
Hprt	Mm00446968_m1
Pdk1	Mm00554306_m1
Pdk2	Mm00446681_m1
Pdk3	Mm00455220_m1
Pdk4	Mm01166879_m1
Hk2	Mm00443385_m1
Ldha	Mm01612132_g1
Pgk1	Mm00435617_m1
