# Supplementary material for: Pyruvate dehydrogenase kinase 1 is essential for transplantable mouse bone marrow hematopoietic stem cell and progenitor function
Source: PLoS One. 2017 Feb 9;12(2):e0171714. doi: 10.1371/journal.pone.0171714 (PMC5300157; doi:10.1371/journal.pone.0171714)
Supplement: S4 Table — (PDF) [file pone.0171714.s007.pdf]

shDNA	Sequence	TRC number
Hif-1α	CCGGCCAGTTACGATTGTGAAGTTACTCGAG TAACTTCACAATCGTAACTGGTTTTTG	TRCN0000054450
Pdk1.11	CCGGGCGGCTTTGTGATTTGTATTACTCGAGT AATACAAATCACAAAGCCGCTTTTTG	TRCN0000078811
Pdk1.12	GGCTTTGTGATTTGTATTATCTCGAGATAATA CAAATCACAAAGCCGTTTTTG	TRCN0000078812
Scramble	CCGGCCTAAGGTTAAGTCGCCCTCGCGAGGG CGACTTAACCTTAGGTTTTG	SHC002
