# Supplementary material for: Pyruvate dehydrogenase kinase 1 is essential for transplantable mouse bone marrow hematopoietic stem cell and progenitor function
Source: PLoS One. 2017 Feb 9;12(2):e0171714. doi: 10.1371/journal.pone.0171714 (PMC5300157; doi:10.1371/journal.pone.0171714)
Supplement: S5 Table — All data generated for results presented in Figs 1–5 are provided as supplementary file containing the raw daya from APT and lactate measurements as well as RT-PCR analyses, and raw data from the fcs-files collected by flow cytometry of transplantation experiments. (DOCX) [file pone.0171714.s008.docx]

**Supporting Information**

**S1 Fig. Gene expression analysis of Pdk4 in muscle, liver, and c-kit^+^Sca-1^+^Lin^-^ (LSK) cells from mouse bone marrow.** qRT-PCR analysis was performed and data were normalized to Hprt expression (n=2-4, in triplicates). Each dot represents one sample, and the data are presented as mean (horizontal line) ± SD. nd, not detected. Pdk4 was undetectable in LSK cells.

**S2 Fig. Hif-1α knockout efficiency analyzed by genotyping.** Genotype analysis of unfractionated BM cells from pIpC–treated Hif-1α^+/+^ and Hif-1α^Δ/Δ^ mice (A) or CFU-GM colonies from one representative Hif-1α^Δ/Δ^ mouse (B). The deleted exon 2 of the Hif-1α gene is indicated by the arrow (300bp).

**S3 Fig. Efficient gene silencing with shRNA to** **Pdk1.** LSK cells were transduced with two different shRNAs to Pdk1 or scramble shRNA as control. Forty-eight hours after transduction, cells were sorted for GFP expression and then incubated for 24 hours in hypoxia or normoxia after which qRT-PCR analysis was performed for expression of Pdk1. The data were normalized to the expression of β-actin (n=3, in triplicates). Each dot represents the mean value of one sample (horizontal line) ± SD. Statistical analysis was performed using a student’s t-test. *, *P* < .05; **, *P* < .01.

**S1 Table. Sequences of primers used in PCR amplification and sequencing reactions.**

**S2 Table. Sequences of primers used in qRT-PCR analysis.**

**S3 Table. Probes used in qRT-PCR analysis.**

**S4 Table**. **shRNA sequences used in the pLKO.1-GFP lentiviral vector.**

**S5 Table. Complete data set Fig. 1-5.** All data generated for results presented in Fig. 1-5 are provided as supplementary file containing the raw daya from APT and lactate measurements as well as RT-PCR analyses, and raw data from the fcs-files collected by flow cytometry of transplantation experiments.
